# The Municipal Control of Milk-supply in Cities and Villages1Presented to the New York State Medical Society.

**Published:** 1898-05

**Authors:** W. H. Heath

**Affiliations:** Buffalo


### THE MUNICIPAL CONTROL OF MILK-SUPPLY IN CITIES AND VILLAGES.[1]

By W. H. Heath, M.D.,
BUFFALO.

Municipal control or supervision of the milk industry in municipalities is, in the present state of affairs, a necessity. It should be comprehensive and made efficient by stringent ordinance, and include scrutiny and vigilance from the dairy to the consumer.

Such measures are justified by the existence of a series of conditions which menace the healthfulness of the product from its source to its distribution; by the fact that it is the most important article of human dietary from infancy to age under all conditions; that, from properties peculiar to it, it is most susceptible to contaminating influences, including bacterial growth; and, finally, by the fact that, notwithstanding its relationship to certain diseases, and especially to the scourge of the race—tuberculosis—the State and the State Board of Health are wofully apathetic or negligent in their attitude toward it.

No time need be occupied before this audience in presenting evidence bearing upon tuberculosis. The contagious character of the disease, its identity in man and the lower animals, its transmissibility from one to the other, as well as the method of restricting it, are now established facts beyond discussion.

It is not assumed that every dairy is an evil. Some few intelligent persons, of up-to-date ideas, are engaged in the business, with suitable and safe equipment; others maintain a high sense of cleanliness, yet there is no uniform or standard procedure of hygiene as there should be. Buildings are in existence illy ventilated, lighted, and cleansed, sodden and soaked with foul material, with bad environment, and where little or no proper care of the herds is given,

[1] Presented to the New York State Medical Society.

and, furthermore, where the work is carried on in such a manner as well as under such conditions that contamination with dirt and by micro-organisms cannot but occur, many of which, like that of tuberculosis and the bacillus coli, are important factors in disease.

A sanitary standard for protection should be adopted by the State under a system of inspection, supervision, and license.

1. It should include the correction of all sanitary evils existing at the present time.

2. Quarantine against the introduction of infected animals from without the State.

3. Veterinary inspection, with the tuberculin-test on all animals, sequestrating those suspected and destroying those infected.

4. A feature to be insisted upon should be the isolation of the herds from other animals, as maladies which attack many of the domesticated animals (chickens, cats, etc.) may, if they do not, carry infection to human beings, and by the milk.

5. Dairy license, issued only after inspection, and subject to revocation upon the violation of proper rules, which should regulate everything pertaining to the business, buildings, herds, employés, methods of milking, cleaning, cooling, and transportation.

Transportation upon railroads in many sections should be likewise scrutinized; protection from the sun and weather at way stations, and refrigeration at terminals insisted upon. The milk should be carried only in cars especially designed for the business, by which is meant those constructed to permit easy and frequent cleaning, not previously used, or used at the same time, for the transportation of deleterious freight, such as decomposing organic matter, vegetable or animal, and additionally equipped with facilities for refrigeration during the heated terms.

Until the existing conditions referred to are remedied the milk supplied the cities is a constant daily source of infection, against which all precautions are justified and should be adopted.

In the absence of State supervision it would seem pertinent to here refer to the system adopted by the Health Commissioner of Buffalo, with the view of mitigating, if possible, the contamination mentioned. By his direction a record of the various dairies supplying the city is kept. This includes inquiry:

First, as to the herds, their health, and whether they are subjected to veterinary examination and tuberculin-test.

Second, the methods of cleaning utensils, the process and time given for cooling the milk, hour of shipment, and distance hauled.

Third, quality and source of the water supply.

Fourth, physical condition of the employés, etc.

Fifth, educational efforts in the way of causing the dissemination of literature bearing upon the interest of the dairyman and his care of the milk.

Following this, where conditions at the dairy are deleterious, or where circumstances arise of a character deemed prejudicial to the public health, instant investigation by the health department is made, and the dairyman required to correct, without delay, such conditions as may be at fault. Failing to do which, or even prior to notification, if urgency and facts demand it, the product of such dairy is interdicted at the city line; the milk-house in the city to which the milk is consigned being at the same time notified and prohibited from receiving the milk, under penalty of the revocation of license.

Under this system, the only one available under the circumstances, it is believed that decided sanitary improvement has been made in the dairies supplying Buffalo, the ratio of infection diminished, and a corresponding influence made upon the public health.

The question of dairies sterilizing their product and shipping it from the dairy sealed, labelled, with date, specific gravity, character, etc., whether pure milk, skimmed milk, or cream, as the case may be, is one that presents many features of advantage, and is worth considering under the present state of affairs.

With this reference to the possible daily pollution or infection of milk before its entry into the city, and the causes permitting it, together with the most practical method of dealing with the problem in the absence of jurisdiction, further protection from bacterial contamination and influences arising from bad sanitation within the city will be best attained by maintaining the following conditions:

Municipal protection could and can be made almost absolute, and in some communities is as efficient and aggressive as State action is negligent and deficient. Municipal ordinance should embody the following features:

1. All milk-houses should be of sufficient and proper size, and should have light and ventilation complying with the rules of hygiene pertaining thereto.

2. They should be constructed of such material as to be sanitary, as much as possible of non-absorbent material, or so made as to permit frequent and effectual flushing and cleansing.

3. Communication, by doors or other openings, with water-closets, sleeping apartments, or other rooms liable to become a source of offence, should be strictly prohibited, nor should milk-rooms be used for any other purposes whatever than the storage of milk.

4. Storage and cooling boxes should be substantially constructed, lined with metal, elevated well above the floors, and away from surrounding walls, to permit thorough cleaning. They should be ventilated into the outer air and properly drained, but no drainage from them should directly communicate with the drainage or plumbing.

5. All plumbing should be strictly sanitary.

6. Rules with specific detailed directions for periodical cleaning of milk-house, storage boxes, cans, bottles, utensils, and information regarding the care of milk and possible sources of contamination should be adopted and conspicuously posted in the milk-room.

7 Cans should be only of such depth and such construction as to permit ready and perfect cleaning, and it should be obligatory that they be cleaned before being returned to the dairy.

8. It should be obligatory that each and every can be tagged at its source, stating the character of the milk, its source, specific gravity as determined at the dairy, with the date and hour of shipment.

9. Milk-rooms should be permitted in cellars only under exceptional circumstances, and then should comply with every requirement of modern hygiene.

10. No milk-house or milk-room should be under the same roof or in connection with any horse or cow stable, nor should it be connected with any unsanitary buildings or surroundings.

11. Construction, equipment, care, and management should be such as to obviate the use of disinfectants, and their presence or use should be prohibited. Any odor or evidence of them about a milk-house should be construed as evidence of defective sanitation.

12. During the summer months no milk should be used when over twenty-four hours old, and during the winter the age of the milk should not exceed thirty-six hours.

13. Milk on the wagon should be thoroughly protected from the weather, and especially during the summer months, by being properly iced, and should be delivered within certain prescribed hours.

14. No milkman should deliver bottles containing milk, nor leave any receptacle of his own containing milk, at any house placarded as containing a contagious disease, nor should he receive any empty bottles or receptacles from such house, or be permitted under any circumstances to fill or refill any bottle while on the wagon.

15. A record of all cases of acute contagious diseases occurring upon the route or in the service of each milkman should be kept. Daily reference and scrutiny of the same should be made, and upon suspicion of relationship of such contagious disease to any one source of the milk-supply, or any one milk-route, immediate investigation and action should follow.

The dealing in milk in small quantities by small shopkeepers, such as grocers, confectioners, and others, should be abolished. Many of these dealers keep milk only as a convenience to their customers. Their facilities for proper storing and handling are insufficient—in fact, nothing. This traffic in milk is objectionable from the fact that it is only a small part of the business, and subsidiary to other interests. The average storekeeper has little or no information relating to milk, which, while in the store, is not only liable to absorb deleterious odors from adjacent products, from vegetables, perchance decomposing, but more seriously to contamination by micro-organisms, from dust and dirt which are tracked in from the street by the feet of customers and blown about the store by the draughts. This condition is always present and a danger, and is invited by the habit of leaving the milk-cans uncovered.

Grocers and other small dealers should be and are obliged, in Buffalo, to keep posted in a conspicuous place the names and license of those from whom they obtain their milk.

To abolish this store peddling will be difficult, but existing conditions should be mitigated by personal instruction to the dealers and frequent inspection by those designated for that duty.

Adulteration of milk by water or contamination by preservation may be considered of secondary importance to the question of sanitation. Stringent ordinances relating to both are proper. Concerning the former, the use of the lactometer as a method of testing has given rise to much discussion through the possibility of evasion at the hands of crafty and unscrupulous dealers. State and city laws upon this feature are liberal, placing the standing as low as it has been deemed possible for normal milk to assume under ordinary conditions.

Vigilance should be exercised to preclude the possibility of water adulteration; though the lactometer is not perfect or absolute, and may be superseded by methods more exact, it is yet a useful instrument, and should be used to discover suspicious cases, which may be subject to further action. Adulteration of milk by water is not a common occurrence in Buffalo, and is seldom found among representative dealers. It is in the main confined to peddlers, who, having an unusual and unexpected demand, resort to the Buffalo water-supply for assistance. (Buffalo water-supply is so good that it is not considered deleterious or dangerous.) The peddling of milk in the streets as well as the distribution of milk through grocery stores and small dealers should properly be abandoned.

The yearly license system, with power of revocation and with heavy penalties for violating ordinances relating to the business, and with the further penalty of exclusion from the trade after a certain number of offences, should be adopted.

The features here specified are necessitated by the fact that in all municipalities the evils they endeavor to cover and prevent have been found to exist, and through their existence contamination has occurred, and disease has been definitely assigned to them. Almost all these directions are, through the direction of Health Commissioner Wende, embodied in the ordinances of the City of Buffalo, and enforced without favor. They have been found efficient, and the security they have given to milk has been demonstrated by the low rate of infantile mortality through diseases known to be due to milk.

In conclusion, features worthy of being incorporated to fortify existing laws relating to the milk industry may be summarized as follows:

1. The method of indirect, though arbitrary, attitude toward the dairies supplying cities, and over which there exists no jurisdiction.

2. The compulsory system of "tagging," giving character, dates, age of milk, prohibiting the sale of milk over twenty-four or thirty-six hours old.

3. Sterilization and preparation at dairy for ultimate delivery, with label system of date and character.

4. Prohibition of disinfectants. Milk-house conditions must preclude their necessity.

5. Protection from infected house by interchange of bottles and receptacles.

6. Record and supervision of the relation of contagious diseases to various milk-routes.

7. The abolition of street peddling and grocery-store dealers.

